# Alternative Splicing Events as Indicators for the Prognosis of Uveal Melanoma

**DOI:** 10.3390/genes11020227

**Published:** 2020-02-21

**Authors:** Qi Wan, Xuan Sang, Lin Jin, Zhichong Wang

**Affiliations:** State Key Laboratory of Ophthalmology, Zhongshan Ophthalmic Center, Sun Yat-Sen University, Guangzhou 510064, China; wanq9@mail2.sysu.edu.cn (Q.W.); sangx@mail2.sysu.edu.cn (X.S.); jinlin3@mail2.sysu.edu.cn (L.J.)

**Keywords:** uveal melanoma, alternative splicing, Lasso, survival analysis

## Abstract

Growing evidence has revealed that abnormal alternative splicing (AS) events are closely related to carcinogenic processes. However, the comprehensive study on the prognostic value of splicing events involved in uveal melanoma (UM) is still lacking. Therefore, splicing data of 80 UM patients were obtained from the Cancer Genome Atlas (TCGA) SpliceSeq and RNA sequence data of UM and patient clinical features were downloaded from the Cancer Genome Atlas (TCGA) database to identify survival related splicing events in UM. As a result, a total of 37996 AS events of 17911 genes in UM were detected, among which 5299 AS events of 3529 genes were significantly associated with UM patients’ survival. Functional enrichment analysis revealed that this survival related splicing genes are corelated with mRNA catabolic process and ribosome pathway. Based on survival related splicing events, seven types of prognostic markers and the final overall prognostic signature could independently predict the overall survival of UM patients. Finally, an 11 spliced gene was identified in the final signature. On the basis of these 11 genes, we constructed a Support Vector Machine (SVM) classifier and evaluated it with leave-one-out cross-validation. The results showed that the 11 genes could determine short- and long-term survival with a predicted accuracy of 97.5%. Besides, the splicing factors and alternative splicing events correlation network was constructed to serve as therapeutic targets for UM treatment. Thus, our study depicts a comprehensive landscape of alternative splicing events in the prognosis of UM. The correlation network and associated pathways would provide additional potential targets for therapy and prognosis.

## 1. Introduction

Uveal melanoma (UM) is the most common type of malignant tumor in adult eyes, and 50% of UM patients will eventually die for their disease [1,2,3]. Although there are certain advances in the diagnosis and treatment of UM, the prognosis for patients with UM remains poor [4]. Therefore, it is important to explore the molecular mechanism underlying the survival events of UM and identify new prognostic factors and therapeutic targets [5].

There have some studies focusing on the prognostic gene signatures of UM, and protein-coding genes of tumor cells are very important in tumor progression [6]. However, due to the limited number of mRNAs, it is difficult to illustrate the complicated function of protein molecular, especially for development and differentiation of tumors [7]. It is well known that alternative splicing (AS) is an important mechanism of post-transcriptional regulation that enables a single gene to produce multiple proteins, and nearly all human protein-coding genes will undergo alternative splicing [8]. Growing evidence has revealed that abnormal splicing events are closely related to carcinogenic processes including angiogenesis, apoptosis, proliferation, metastasis, and therapeutic drug resistance [9]. Thus, many cancer investigators are aware of the significance of AS and believe that it can take a potential role in therapeutic target. Evidence has demonstrated that AS serves an important role in the occurrence and development of cancers. For instance, splicing events happen in CD44, Ron and MENA will lead to cellular proliferation, VEGF causes angiogenesis, Fas, Bclx, and caspase-2 result in apoptosis and p53 brings about multi-drug resistance [9]. Generally, the complex process of AS is dynamically regulated by limited splicing factors. Abnormal expression of splicing factors may lead to whole changes of AS events in tumors and in particular situation, and the mutations of splicing factor can cause specific splicing patterns to promote cancer progression [10,11]. Therefore, recent studies have paid more attention to assessing the clinical significance of AS events and splicing factors in cancers and their potential pathogenic pathways and regulatory networks.

Although there is an increasing systematic analyses of prognostic AS signatures in colorectal cancer, glioblastoma, lung cancer breast cancer, and ovarian cancer [12,13,14,15], the comprehensive study for the prognostic value of splicing events involved in UM is still lacking. Fortunately, the splicing data of UM is currently available through the Cancer Genome Atlas (TCGA) SpliceSeq website, which can be used to gain numerous of splicing events to predict the prognosis of UM. 

Hence, in this study, we systematically evaluated the expression of alternative splicing events in 80 UM samples from the Cancer Genome Atlas (TCGA) dataset [16]. We identified some survival related AS events and biological pathways. More importantly, we identified am 11 spliced gene prognostic marker that could significantly predict UM survival and constructed a splicing network that could evaluated their potential functions in tumor biology.

## 2. Material and Methods

### 2.1. Raw Data Process

**Figure 1:**
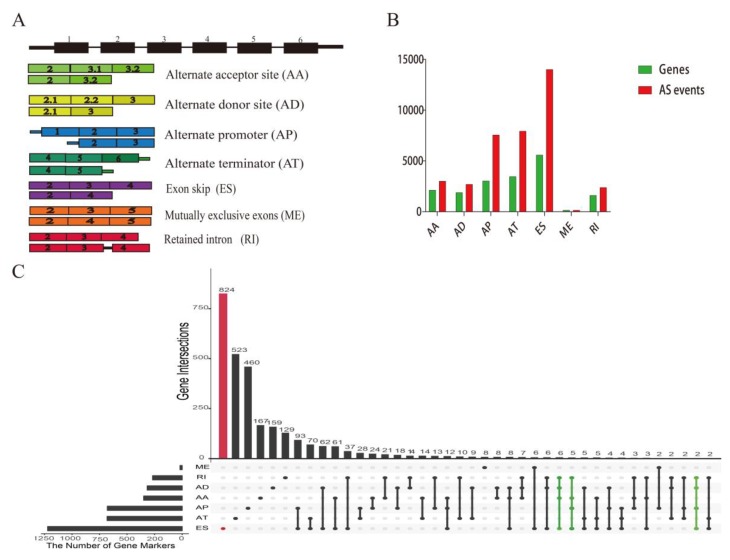
Landscape of alternative splicing (AS) events in uveal melanoma (UM). AA, alternate acceptor; AD, alternate donor; AP, alternate promoter; AT, alternate terminator; ES, exon skip; ME, mutually exclusive exons; RI, retained intron. (**A**) Illustration for seven types of alternative splicing in this study. (**B**) The number of AS events and corresponding genes included in the present study; the x-axis stands for the types of alternative splicing, and y-axis means the number of genes and AS events. (**C**) UpSet plot of different types of alternative splicing types in UM. The dark bar on the left of drawing represents the amount of each type of AS event. The dark dots in the matrix at the right of drawing represent the intersections of AS events. One gene might possess several alternative splicing patterns (dark dot line), and even a single gene can undergo four types of alternative splicing (green dot line). ES was the most common type of the alternative splicing events (red bar).

The mRNA data and corresponding clinical information of UM were downloaded from TCGA (https://tcga-data.nci.nih.gov/). This dataset was derived from the tissue samples of 80 adult patients. Alternative splicing data of UM were obtained from TCGA SpliceSeq (https://bioinformatics.mdanderson.org/TCGASpliceSeq) [17]. In TCGA SpliceSeq, a value called Percent Spliced In (PSI), ranging from 0 to 1, was calculated for each detected AS event in a gene. These alternative splicing events can be classified into seven patterns, including the Alternate Donor site (AD), Alternate Acceptor site (AA), Alternate Terminator (AT), Alternate Promoter (AP), Mutually Exclusive Exons (ME), Exon Skip (ES), and Retained Intron (RI). The illustration of the seven types was visualized in Figure 1A. For each sample and every possible splice event, a PSI value was calculated, which suggests a shift in splicing events. AS events with a PSI value of ≥75% and ΔPSI ≥ 30% in the UM cohort samples were selected for further research. 

### 2.2. Identification of Survival-Related Alternative Splicing Events

The overall survival time of UM was also downloaded from TCGA. In order to screen survival-related AS events, we classified patients into two groups by the median PSI value of each splicing event and performed univariable cox regression analysis. Splicing events expressing significance *p* values < 0.05 were selected as survival-related AS.

### 2.3. Construction of the Spliced Gene Correlation Network

To evaluate the interactive relationships among survival-related alternative spliced genes, correlation analyses of survival-related spliced genes was applied by using STRING database which is widely used to predict protein-protein interactions (http://stringdb.org). Moreover, the functions of significant survival-related spliced genes were performed by the bioinformatic tool “clusterProfiler” (Guangchuang YU, Southern Medical University, Guangzhou, China).Gene ontology (GO) functional enrichment analyses and Kyoto encyclopedia of genes and genomes (KEGG) pathways were used to assess the functional categories, which consists of an integrated biological knowledge base and analytic tools aimed at systematically extracting biological meaning from large gene/protein lists. Adjust *p* value < 0.05 were considered to be significant.

### 2.4. LASSO Multivariate Cox Analysis

The PSI values of survival-related spliced genes were obtained as described above. Next, the LASSO was used to construct prognostic markers with selected survival-related spliced genes. According to the expression level of each sample, LASSO determines the qualified spliced genes for the risk system and generates the risk score of each sample. The patients were divided into high-risk group and low-risk group by using the median cutoff risk score. Subgroups analysis of somatic mutation and clinical characteristics were evaluated. The survival curves of Kaplan-Meier were drawn, and the differences among groups were compared by log-rank tests. In addition, in order to assess the specificity and sensitivity of gene signature, receiver operating characteristic (ROC) curves for predicting overall survival (OS) were drawn, and area under the curve (AUC) values were generated. In addition, an SVM classifier was constructed based on the final selected survival-related spliced genes, and its performance was evaluated by leave-one-out cross-validation.

### 2.5. Construction of Splicing Factor Correlation Network

In order to assess the relationships between splicing factors and prognostic alternative splicing events. We first selected 119 splicing factor genes from previously published article and then the expression of splicing factor genes was screened from RNA-seq data of UM [18]. Kaplan–Meier survival analysis was also applied to evaluate the survival associated splicing factor genes. Spearman analyses was performed to explore the correlation between the survival associated splicing factor genes and the survival-related AS events. *p* < 0.05 was considered significant, and the correlation plots were visualized by Cytoscape.

### 2.6. Statistical Analysis

All statistical analyses were conducted using R software (v.3.5.2). The intersection of seven AS patterns was applied by using the “UpSetR” package. Survival analysis were performed by using “survival” and “survivalROC” package. LASSO multivariate cox analysis was calculated with the “glmnet” package. SVM method was conducted by “e1017” package. The correlation coefficient was calculated by Spearman test. The Kaplan–Meier survival analysis was applied to compare the overall survival of the patients in the different groups or in the low- and high-risk groups. *p* < 0.05 was regarded as statistically significant in all statistical tests.

## 3. Results

### 3.1. Alternative Splicing Events in UM

As a result, a total of 37996 AS events in 17,911 genes in UM were detected, which included 5599 ESs in 14,019 genes, 1619 RIs in 2388 genes, 2130 AAs in 3022 genes, 1891 ADs in 2705 genes, 3048 APs in 7565 genes, 3465 ATs in 7938 genes, and 159 MEs in 159 genes (Figure 1B). These results also revealed that one gene may have multiple types of alternative splicing events. Even one gene might undergo up to four types of alternative splicing. Among types of AS events, ES was the most frequent type of the alternative splicing events and the infrequent types were ME events (Figure 1C).

### 3.2. Identification of Survival-Related Alternative Splicing Events

**Figure 2:**
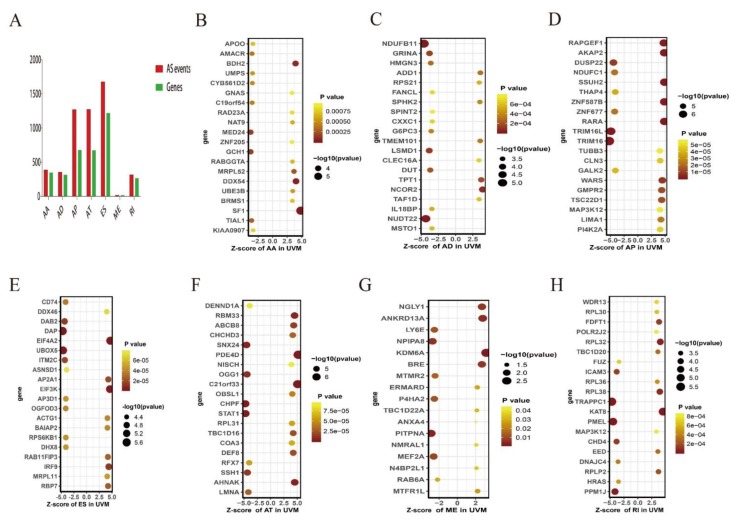
The 20 most significant AS events in UM. (**A**) The number of survival-related AS events and corresponding genes obtained by using univariable cox regression analysis. The x-axis stands for the types of alternative splicing, and the y-axis stands for the number of genes and AS events. (**B–H**) Forest plots of the top 20 significantly survival-related AS events for acceptor sites, the x-axis stands for z-scores, and the y-axis stands for survival-related AS events: (**B**) AA, alternate acceptors; (**C**) AD, alternate donors; (**D**) AP, alternate promoters; (**E**) ES, exon skip; (**F**) AT, alternate terminators; (**G**) ME, mutually exclusive exons; (**H**) RI, retained introns.

In total, 387 AAs in 349 genes, 354 ADs in 318 genes, 1271 APs in 679 genes, 1274 ATs in 676 genes, 1678 ESs in 1221 genes, 18 MEs in 18 genes, and 317 RIs in 268 genes were identified as prognosis-associated AS events (*p* < 0.05) (Figure 2A). The 20 most significant survival-related alternative splicing events in UM are shown in Figure 2B–H. Among them, there were only 18 prognostic ME events.

### 3.3. Construction of Spliced Gene Correlation Network

**Figure 3:**
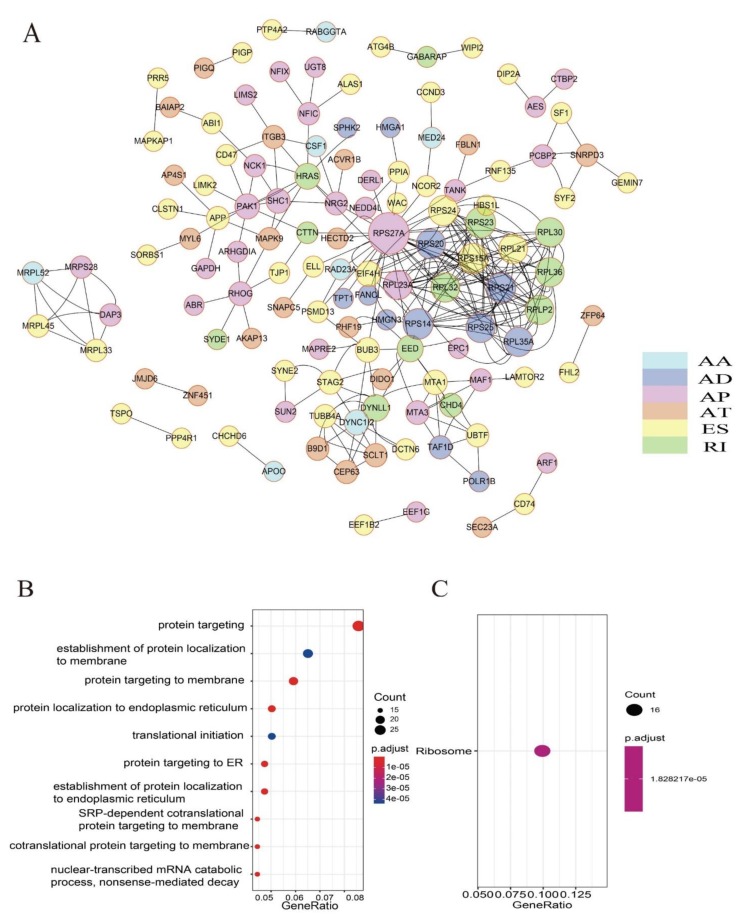
Interaction network of survival-related alternative splicing (AS) events. (**A**) Protein–protein interaction network of genes with survival-related AS events in UM. (**B**) Gene ontology (GO) analysis of genes with survival-related AS events. The x-axis stands for gene ration in the background gene, and the y-axis stands for the term of pathway. (**C**) KEGG pathway analysis of genes with survival-related AS events. The x-axis stands for gene ratio in the background gene, and the y-axis stands for the term of pathway.

Finally, there are 383 significant survival-related spliced genes in UM (*p* < 0.001) mapped to STRING database, and Cytoscape was used to visualize the gene correlation network (Figure 3A; the cytoscape file is provided as Supplementary Material). Furthermore, in order to explore the molecular functions of genes with survival-related alternative splicing events. The “clusterProfiler” method was applied to annotate and the results showed that 21 pathways were significantly enriched in biological process terms including “protein targeting,” “protein targeting to membrane,” “mRNA catabolic process,” “nuclear-transcribed mRNA catabolic process,” etc. (Figure 3B). In addition, we found that only “Ribosome” pathway was significantly correlated with these spliced genes in KEGG terms (Figure 3C).

### 3.4. LASSO Analysis Based on Selected Splicing Events

**Figure 4:**
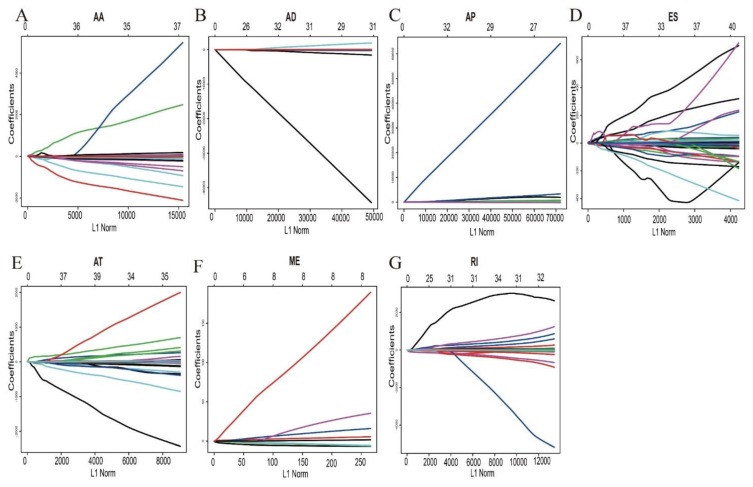
Construction of seven splicing types of prognostic markers based on least absolute shrinkage and selection operator (LASSO) analysis. LASSO coefficient profiles of survival-related alternative splicing (AS) events: (**A**) AA, alternate acceptors; (**B**) AD, alternate donors; (**C**) AP, alternate promoters; (**D**) ES, exon skip; (**E**) AT, alternate terminators; (**F**) ME, mutually exclusive exons; (**G**) RI, retained introns. The lines stand for each survival-related AS events in seven splicing types respectively and candidate AS events were selected by using 10 fold cross-validation via minimum criteria; X-axis is stand for LASSO coefficient profiles of survival-related alternative splicing (AS) events. Y-axis means tuning parameter (lambda) selection in the Lasso regression.

**Figure 5:**
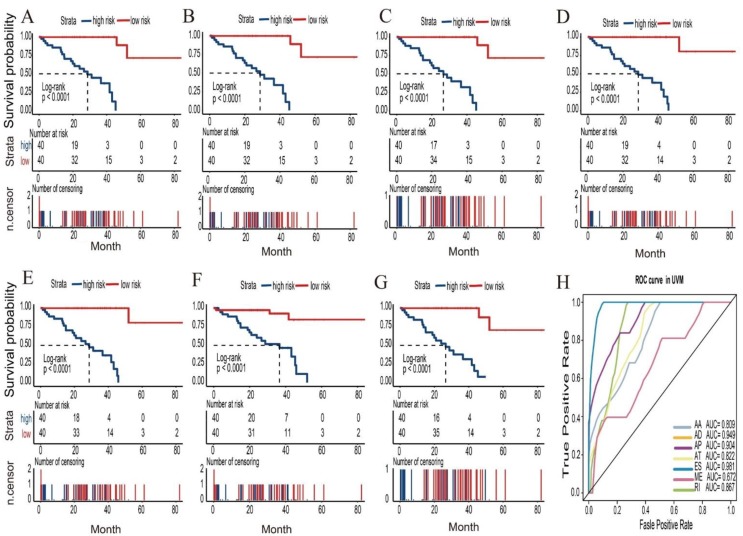
Kaplan–Meier plots and receiver operating characteristic (ROC) curves of predictive factors in UM cohort. (**A**–**G**) Kaplan–Meier curves of prognostic models built with alternative splicing (AS) events of alternate acceptor (AA), alternate donor (AD), alternate promoter (AP), alternate terminator (AT), exon skip (ES), retained intron (RI), and mutually exclusive exon (ME) splicing types for patients with UM. Time-dependent numbers at risk are listed at the middle panels and the number of censor patients are listed at the bottom panels. (**H**) The ROC curves of predictive models for each splicing type in UM.

**Figure 6:**
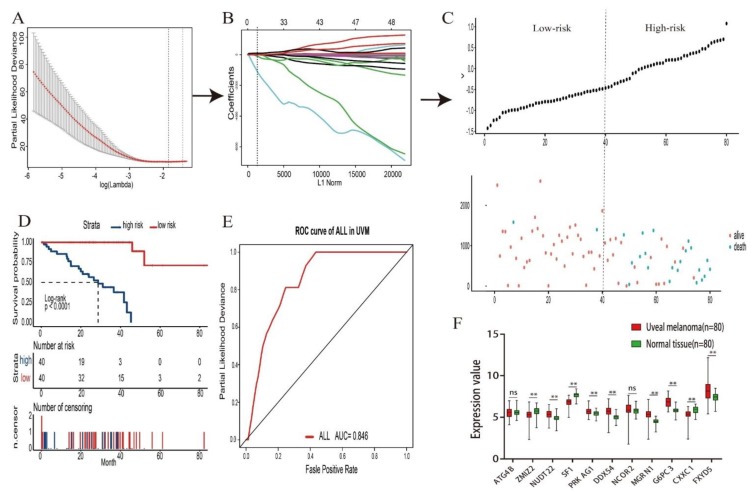
The final overall prognostic model in UM. (**A**–**C**) The process of building the signature containing all survival-related AS events and the coefficients calculated by LASSO method: (**A**) Partial likelihood distribution with the corresponding λ-logarithm value and the left variants of model (**B**) LASSO coefficient profiles of all survival-related alternative splicing (AS) events. A vertical line is drawn at the value chosen by 10-fold cross-validation. (**C**) The distribution of risk score, overall survival (OS) and life status for the 80 patients in UM. (**D**) Kaplan–Meier overall survival curves of the final prognostic model. Time-dependent numbers at risk are listed at the middle panel and the number of censor patients are listed at the bottom panel. (**E**) The ROC curves of predictive model for all splicing types in UM. (**F**) The expression values of 11 spliced genes in UM and healthy normal tissue (** *p* < 0.01, ns means no significance).

**Figure 7:**
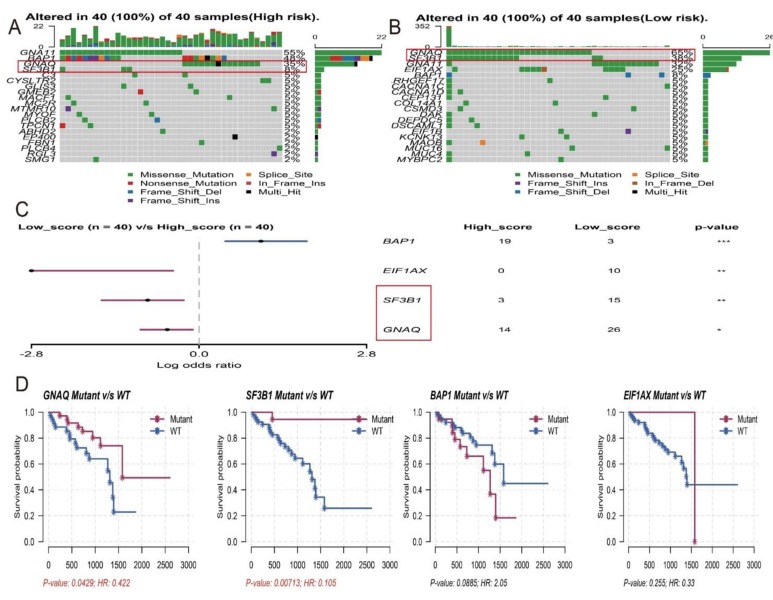
Differential landscape of somatic mutation burden between high and low risk groups. (**A**) The waterfall plots of the top 20 mutant genes in high risk group. (**B**) The waterfall plots of top 20 mutant genes in low risk group. (**C**) Forestplot suggested that three genes GNAQ, SF3B1 and EIF1AX which are highly mutated in low-risk group compared to high-risk group (*** *p* < 0.001, ** *p* < 0.01, * *p* < 0.05). (D) Kaplan–Meier overall survival curves of four different mutated genes. The mutant of GNAQ and SF3B1 have a longer survival time than the wild type. With log-rank P = 0.043 and log-rank P = 0.007 respectively.

In order to build prognostic markers for UM patients, the least absolute shrinkage and selection operator (LASSO) cox analysis was used to construct prognostic markers with selected survival-related AS events. Then, we constructed seven types of prognostic markers based on AA, AD, AP, AT, ES, ME, and RI, respectively (Figure 4). Interestingly, these seven prognostic markers can independently predict the overall survival of UM patients (Figure 5A–F). The area under cure (AUC) values of seven types were 0.809, 0.949, 0.904, 0.822, 0.981, 0.672, and 0.867, respectively, which showed a good performance in prognosis prediction (Figure 5H). Moreover, we selected all the significant survival-related splicing events in the seven types and develop an 11 spliced gene prognostic marker (Figure 6C). The risk system was built and reckoned a risk score for each patient. Applying the medium cut-off value of the risk scores. Eighty UM patients were divided into high-risk and low-risk groups (Figure 6C). The expression value of 11 spliced genes in UM and normal samples which come from GTEx (Genotype-Tissue Expression) database were showed in Figure 6F as well. The results indicated that most of genes were markedly different in the tumor and normal samples. Kaplan-Meier curve indicated that there is a significant difference between high-risk and low-risk group with log-rank test of *p* < 0.0001 (Figure 6D). The ROC curve indicated that the final 11 spliced gene prognostic marker could significantly and robustly predict UM survival (Figure 6E). What’s more, we tried different number of selected survival-related spliced genes and calculated their prediction performance. On the basis of these performances, we plotted an accuracy curve (shown in Figure S1). Finally, the 11 spliced genes could determine short- and long-term survival with a predicted accuracy of 97.5% (Table 1). The subgroups analysis of clinical characteristics between low- and high- risk groups showed that vital status, age, histological type and living time have a significant difference (Table 2). The forestplot showed three genes GNAQ, SF3B1 and EIF1AX which are highly mutated in low-risk group compared to high-risk group (Figure 7A–C). The mutant of GNAQ and SF3B1 also indicated a significantly longer OS time than the wild type (Figure 7D).

### 3.5. Construction of Splicing Factor Correlation Network

**Figure 8:**
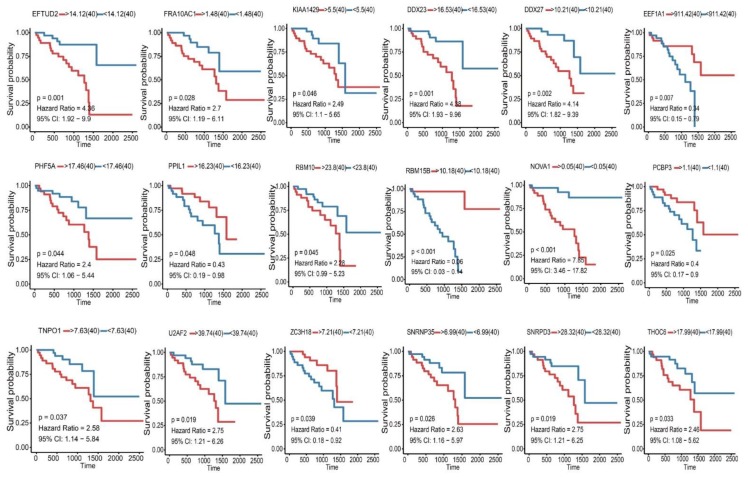
Kaplan-Meier survival analysis for 18 survival-associated splicing factor genes of UM. Their expression levels were classified into two groups by median value. Blue, low-level group; red, high-level group.

**Figure 9:**
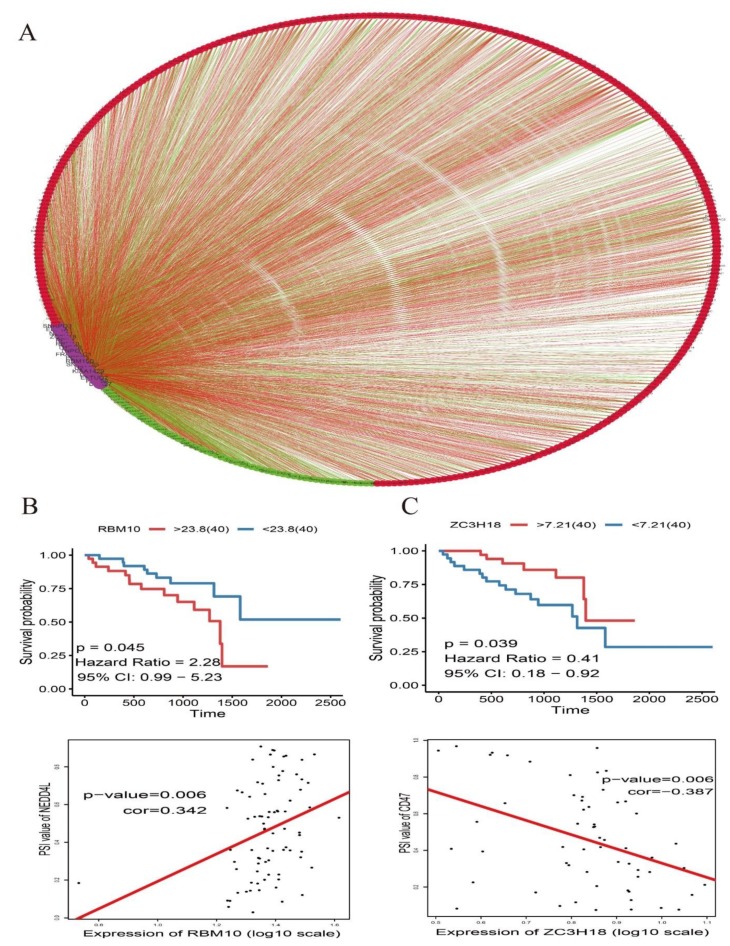
Survival-associated splicing factors and splicing correlation network in UM. (**A**) Splicing correlation network in UM patients constructed by Cytoscape. Eighteen survival-associated splicing factors (purple dots) were positively (red lines) or negatively (green lines) associated with AS events, which predicted good (green dots) or poor (red dots) outcomes in UM patients. (**B**) High RBM10 expression was significantly associated with poor overall survival in UM. Positive correlations between RBM10 expression and the Percent Spliced In (PSI) value of NEDD4L. (**C**) Low ZC3H18 expression was significantly associated with poor overall survival in UM. Negative correlations between ZC3H18 expression and the PSI value of CD47.

First, the expression of 119 splicing factor genes was screened from RNA-seq data of UM. Kaplan-Meier survival analysis revealed that 18 splicing factor genes whose expression levels were significantly associated with overall survival (Figure 8). Next, the Spearman test was used to evaluate the correlations between survival-associated splicing factors and alternative splicing events, and correlation networks were constructed using Cytoscape (cytoscape file to be provided as Supplementary Material). In UM patients, a total of 3871 alternative splicing events were significantly associated with splicing factors, with 701 positively (red lines) and 3170 negatively (green lines) related alternative splicing events (Figure 9).

## 4. Discussion

Alternative splicing is involved in the regulation of almost all multiexons. Abnormal alternative splicing is widely regarded as a new indicator of carcinogenic processes [19,20]. Although some specific mRNAs related to alternative splicing of UM have been identified: For instance, SF3B1 mutations can cause differential alternative splicing of protein coding genes and the aberrant of CD44 alternative splicing are commonly existed in UM [21,22,23,24]. The exploration of alternative splicing events and their related pathways in UM is far from comprehensive. With the growing development of high-throughput sequencing and bioinformatic methods, (TCGA) SpliceSeq (http://bioinformatics.mdanderson.org/TCGASpliceSeq) has emerged and could be used to gain numerous profiles to comprehensive overview of alternative splicing events in UM. In the current work, we conducted our research with the help of numerous computational tools and public data like TCGA. A series of survival-associated alternative splicing events was identified, and their interaction network was also established, which could provide a novel intervention target for UM treatment.

Different splicing patterns cause one gene to produce multiple subtypes, which makes alternative splicing more complex in the regulatory mechanism of cancer development. In our research, the most frequent alternative splicing pattern is ES which is consistent with many other cancer researches [8,9,11]. A total of 523 alternative splicing events in 383 genes were regarded as the top (*p* < 0.001) significant survival-related splicing events. In the spliced gene correlation network, the family of ribosomal protein genes RPS27A, RPS14, RPS23, RPS20, RPS24, RPS15A, RPS21, and RPS25 were the hub genes. Remarkably, it is widely accepted that defects in the ribosome are associated with cancer [25]. Recent studies have revealed that alternative splicing in ribosomal protein genes were identified in leukemias and solid tumor types. In this paper, we also identified some potential new biological pathways associated these survival-related spliced genes like mRNA catabolic process, nuclear-transcribed mRNA catabolic process and ribosome. These findings also point out the way for future clinical application. As we known, ribosome biogenesis is a tightly regulated process that is critical for fundamental cellular functions including cell growth and division [26]. These ribosomal protein genes which encode structural proteins associated with ribosome biosynthesis are the most suitable for treatment in UM.

In addition, in order to refine the prediction model and help specify the predicted value of alternative splicing patterns in UM, we performed an ROC curve analysis and the LASSO method to build prediction models. Interestingly, these seven types of alternative splicing markers revealed a good overall survival prediction of UM. The AUCs of prediction models for each splice type were different from each other, with the best AUC values being 0.981 in ES predictive model and the poor AUC values being 0.672 in ME predictive model, respectively. Furthermore, the final prediction model of all splice type suggested that an 11 spliced gene prognostic marker can better to predict the prognosis of UM with AUC value of 0.846. These 11 spliced genes including MGRN1, ATG4B, SF1, DDX54, NCOR2, CXXC1, FXYD5, ZMIZ2, NUDT22, G6PC3 and PRKAG1. Stratified analysis revealed that this 11 spliced gene signature can further stratify UM patients into subgroups with different mutation patterns and was proper for predicting the vital status, age and histological type of UM. Obviously, the performance needed to be validated in an independent large dataset, but the in-depth biological analysis of these biomarkers and compared the mRNA expression to normal samples showed a great promise and suggested that these biomarkers played important roles in UM [27]. To sum up, these alternative splicing prediction models can provide new prognostic information to promote the prognosis evaluation of UM patients. 

Alternative splicing events are primarily regulated by splicing factor genes through affecting the binding of exons selection and splicing sites [28]. Therefore, exploration of the splicing factors and alternative splicing events correlation network is imperative in UM. Here we identified 18 splicing factors were significantly co-related to UM patients’ survival (Figure 7), such as RBM10 and ZC3H18. RBM10 (RNA-binding protein 10) is belongs to RNA binding proteins family and regards as a tumor promoter universally participate in tumorigenesis by influencing tumor proliferation and apoptosis [29]. For example, recent researches revealed that high expression of RBM10 protein in lung cancer was associated with a shorter overall survival time and a poor prognosis [30]. Interestingly, our result consistent with this conclusion (Figure 9B). Furthermore, the expression of RBM10 is positive co-related with the expression of NEDD4L. Yusuke Kito et al. reported that NEDD4L expression may be increased to facilitate tumor growth in many melanomas [31]. As for ZC3H18 (Zinc finger CCCH domain-containing protein 18), our study suggested that the gene of ZC3H18 could be regarded as a protective factor and negatively associated with CD47 (Figure 9C). Massive studies have shown that overexpression of CD47 predicts poor prognosis and promotes cancer cell invasion in many types of cancer [32,33,34]. What is more, in this correlation network, we found most of alternative splicing events were associated with poor overall survival and positively co-related to splicing factors. It is reasonable to believe that there is an obvious trend for the expression of splicing factors are positively associated with the poor prognostic alternative splicing events in UM. Thus, this phenomenon suggested that up-regulation of splicing factors and down-regulation of splicing factors may serving as therapeutic targets for UM treatment.

Although we identify some significant AS events and spliced genes for prognostic of UM, our research still has some limitations. The research is based on bioinformatics method and its conclusion has not been confirmed by experiments. In addition, the sample size in our study was limited. Therefore, it is necessary to do more work to further explore this molecular mechanism.

To sum up, our study depicts a comprehensive landscape of alternative splicing events in UM and identified that survival-related alternative splicing signatures can be used to predict overall survival of UM patients. Prognostic splicing factors and an alternative splicing events network were constructed in UM to present a whole picture of the expression interactions, which revealed a novel underlying mechanism in the tumorigenesis of UM. Further investigations are needed to reveal the clinical and biological significance of the non-cancer cells and genes of alternative splicing events in U, so as to better guide the more effective diagnosis and prognosis of UM.

## Figures and Tables

**Table 1:** The confusion matrix of the predicted and actual sample classes.

	Actual Long-Term Survival	Actual Short-Term Survival
Predicted long-term survival	38	0
Predicted short-term survival	2	40
Sensitivity: 0.950	Specificity: 1	Accuracy: 0.975

**Table 2:** The subgroups analysis of clinical characteristics between low- and high- risk groups. The results indicated that UM patients in high-risk have older age, shorter living time and more epithelioid cell type than low-risk (chi-square test *p* < 0.05).

	High-Risk	Low-Risk	*p*
n	40	40	
vital_status = DEAD (%)	21 (52.5)	2 (5.0)	<0.001
Race = white (%)	25 (100.0)	30 (100.0)	NA
Age (mean(SD))	67.26 (14.10)	60.30 (13.91)	0.029
Gender = MALE (%)	26 (65.0)	19 (47.5)	0.176
Stage (%)			0.148
	1 (2.5)	0 (0.0)	
StageII	18 (45.0)	21 (52.5)	
StageIII	17 (42.5)	19 (47.5)	
StageIV	4 (10.0)	0 (0.0)	
m (%)			0.22
m0	25 (64.1)	26 (66.7)	
m1	2 (5.1)	0 (0.0)	
m1b	2 (5.1)	0 (0.0)	
mx	10 (25.6)	13 (33.3)	
n = nx (%)	13 (33.3)	14 (35.0)	1
t (%)			0.4
t2a	3 (7.5)	9 (22.5)	
t2b	1 (2.5)	1 (2.5)	
t3	1 (2.5)	0 (0.0)	
t3a	14 (35.0)	11 (27.5)	
t3b	1 (2.5)	4 (10.0)	
t3c	1 (2.5)	0 (0.0)	
t4a	11 (27.5)	9 (22.5)	
t4b	4 (10.0)	5 (12.5)	
t4c	1 (2.5)	1 (2.5)	
t4d	2 (5.0)	0 (0.0)	
t4e	1 (2.5)	0 (0.0)	
histological_type (%)			0.003
EpithelioidCell	10 (25.0)	3 (7.5)	
SpindleCell	8 (20.0)	22 (55.0)	
SpindleCell|EpithelioidCell	22 (55.0)	15 (37.5)	
age_group = younger (%)	17 (42.5)	23 (57.5)	0.264
time (mean (SD))	11.83 (9.94)	18.58 (17.40)	0.036
AGE => 60 (%)	28 (70.0)	19 (47.5)	0.069

## Supplementary Materials

The following are available online at https://www.mdpi.com/2073-4425/11/2/227/s1, Figure S1: The accuracy curve with the number of genes and the performance of classifiers. The x-axis was the number of genes used for SVM classifier construction and the y-axis was the accuracy of the SVM classifier evaluated with Leave one out cross validation (LOOCV). The peak of curve was accuracy of 0.97 when 11 spliced genes were used.
