# Supplementary material for: Alternative Splicing Events as Indicators for the Prognosis of Uveal Melanoma
Source: Genes (Basel). 2020 Feb 21;11(2):227. doi: 10.3390/genes11020227 (PMC7074237; doi:10.3390/genes11020227)
Supplement: Supplementary file 1 [file genes-11-00227-s001.zip › genes-708089 Figure S1.pdf]

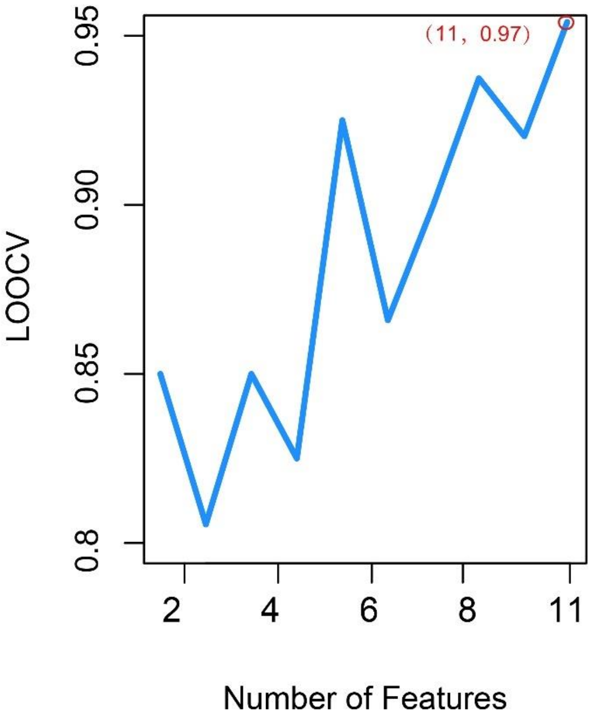

Supplemental Figure 1. The accuracy curve with the number of genes and the performance of classifiers. The x-axis was the number of genes used for SVM classifier construction and the y-axis was the accuracy of the SVM classifier evaluated with Leave one out cross validation (LOOCV). The peak of curve was accuracy of 0.97 when 11 spliced genes were used.
